# Supplementary material for: Dynalign II: common secondary structure prediction for RNA homologs with domain insertions
Source: Nucleic Acids Res. 2014 Nov 21;42(22):13939–48. doi: 10.1093/nar/gku1172 (PMC4267632; doi:10.1093/nar/gku1172)
Supplement: SUPPLEMENTARY DATA [file supp_gku1172_nar-02021-z-2014-File012.zip › manual/Developer/SWIG.doc]

**RNAstructure**

**Developer's Manual**

**SWIG**

**Version 1.0: 09/28/2012**

**Table of Contents**

Overview

SWIG Interface Files

The Structure Drawing Proxy

The Dot Plot Drawing Proxy

The RNAstructure Interface Proxy

Programming Example

**Overview**

All Java classes in the RNAstructure repository that are backed by C++ classes are linked to C++ using a program called SWIG (Simplified Wrapper Interface Generator). SWIG generates C++ and Java classes that can link together using the Java Native Interface (JNI).

For each Java Makefile in the repository (RNAstructure_java_drawing/Makefile and RNAstructure_java_interface/Makefile), SWIG commands can be found under the swigBuild target. SWIG is not necessary to run the Java components of the repository, only to build them. Even then, building SWIG is usually not necessary unless method signatures in the C++ proxy classes backing the Java classes have been changed. This is because the SWIG-specific files are committed to the cvs repository. The specific files generated by SWIG will be detailed in later sections. A rebuild would be necessary if methods were added to the C++ proxy classes or the parameter list in one method was changed. A rebuild would not be necessary, however, if the way a method worked was changed, but its method signature and return type remained the same.

To build a SWIG proxy class, use the following general syntax:

swig -java -c++ -package <pkgname> -outdir <dirname> swigFile.i

rm -f <dirname>/*Proxy.java

In the first line, it says to run SWIG (swig) to get Java (-java) from C++ (-c++). Identify the Java files as being in a particular package (-package <pkgname>) and put them in a particular output directory (-outdir <dirname>). Use the given SWIG interface file (swigFile.i) to do this.

In the second line, it says to remove all files from the output directory that end in Proxy.java. This is because those classes are automatically generated by SWIG, but they are empty and not useful for our purposes, so they can be safely deleted.

General information describing how SWIG interface files are made can be found on the SWIG web site: [http://www.swig.org](http://www.swig.org/). The interface files used to create proxies in the RNAstructure repository will be explained in a later section.

All C++ header files that are to be wrapped by SWIG or included in interface files must include the standard C++ namespace (using namespace std;). This isn't always necessary for all SWIG wrapping, but for our purposes it is, since so many of our back end classes use the namespace.

When creating a class to be wrapped with SWIG, it is essential that method return types and parameters always be either simple number types (byte, short, int, float, double, long), or strings. Note that this means the string object from the <string> class, NOT const char* or char*. Keeping parameters and return types simple like this ensures separation of front end and back end data, and leaves all memory management issues on the C++ side. Managing memory through the JNI is very difficult to get right, and is not recommended.

If strings are used in a SWIG proxy, the SWIG helper file std_string.i must be included in the interface file.

It is best to put as much error checking as possible in the C++ proxy class. Always make sure that Java has a way to access what kind of error happened, if one did occur.

NEVER use inheritance when wrapping a class for SWIG. There are bugs in the program that cause the generated code to be wrong, which cause segmentation faults on the C++ side and virtual machine crashes on the Java side. When the Java virtual machine crashes in this way, the Java program terminates, and a detailed error is printed out to standard error. Usually, the error says something similar to the following:

\# A fatal error has been detected by the Java Runtime Environment:

\#

\# SIGSEGV (0xb) at pc=0x00007f0b024734cd, pid=21947, tid=139676677560592

\#

\# JRE version: 6.0_15-b03

\# Java VM: Java HotSpot(TM) 64-Bit Server VM (14.1-b02 mixed mode linux-amd64 )

\# Problematic frame:

\# C [libRNAstructure_GUI.so+0x5df4cd]

This error means that a segmentation fault has occurred somewhere in native code. Although the exact method cannot be determined from the above example (it is a little contrived), usually the top problematic frame in the error message will reveal which method caused the segmentation fault. The pid shown in the error above is an error file identification number; usually a file called hs<pid>.log (in this case hs21947.log), will be placed in the directory from which the application was run. This file contains a stack trace that gives more detail about where the segmentation fault came from. Usually, however, all this extra data is not necessary for debugging.

A C++ SWIG proxy class should be a stand-alone class, i.e. outside of any type of inheritance hierarchy, that is not used for anything else besides a proxy. It can include as many classes and types of calculations as necessary.

In order for the SWIG wrapped class to be successfully used in Java, it must be compiled and linked into a dynamic library. Java has a standard method called System.loadLibrary() that knows the proper syntax to look for in a library name, as long as it is given a proper identifier. For example, consider the following line:

System.loadLibrary( "RNAstructure_GUI" );

This line is used to identify the dynamic library in the RNAstructure Java interface. Java will look for "libRNAstructure_GUI.so" on a Linux/Unix system, "libRNAstructure_GUI.dylib" on a Macintosh system, and "RNAstructure_GUI.dll" on a Windows system. The method is smart enough to know what kind of operating system it's being used on, and looks for the appropriate dynamic library name.

As long as the SWIG proxy and all C++ source code files used within it compile and link properly into this dynamic library, the C++ code will be accessible to Java through the dynamic library and SWIG proxy class calls.

**SWIG Interface Files**

All SWIG interface files used in the RNAstructure repository look similar, and all follow the same basic pattern in form. The repository contains three SWIG proxy classes: DotPlotBackend, StructureBackend, and RNAstructureBackendCalculator. Each of these has a SWIG interface file similarly named. So, for a given SWIG interface file called <name>.i (note that SWIG interface files always must have a .i extension), the format of the interface file is as follows:

```
%module <name>Proxy;

%{
#include "<name>.h"
%}

%include "std_string.i"

%include "<name>.h"
```

The first line declares the SWIG file's module name. The module name is not important for our purposes, and the file generated for the module (<name>Proxy.java) should be deleted after SWIG runs. For more information on modules, see the SWIG website referenced in the overview section.

The second group of three lines is a C++ include statement for the header file of the class that is being wrapped, surrounded by special SWIG brackets. Any text inside these brackets is placed verbatim into the generated C++ proxy file. For simplicity, the only thing we want there is an include statement, to make sure the proxy file knows what it is wrapping.

Since all the SWIG proxy classes use the C++ string object (rather than const char*), the SWIG helper file std_string.i is included in the interface file. This tells the proxy files generator that provisions need to be made to allow Java strings to be converted into C++ strings and vice versa. The conversion works best when the Java String class and the C++ string class are used (Note that in Java, the class is capitalized, while in C++, it is not.), so character arrays in place of strings should be avoided in both the Java and C++ proxies.

On the last line, the C++ header file is placed in front of the SWIG include directive. This line tells the SWIG generator which methods are being wrapped in the header file – all of them.

There are no comments in the SWIG interface files because the SWIG commenting system is currently deprecated. As of this writing, there are plans to reinstate it in future releases of SWIG, but it probably won't be necessary for these simple interface files.

Any further information, if more complex interface files need to be created in the future, can be found on the SWIG website.

**The Structure Drawing Proxy**

The structure drawing proxy consists of the following files. The first four can be found in RNAstructure_java_drawing/SWIG, and the last two can be found in RNAstructure_java_drawing/source/proxy. The first three are written by the developer, and the last three are generated by SWIG.

1. StructureBackend.i (SWIG interface file)
2. StructureBackend.h (C++ header file for SWIG wrapped class)
3. StructureBackend.cpp (C++ implementation file for SWIG wrapped class)
4. StructureBackend_wrap.cxx (C++ proxy file generated by SWIG)
5. StructureBackend.java (Java class that is used to access wrapped C++)
6. StructureBackendProxyJNI.java (Java class that calls native methods from the wrapper class)

Of all these classes, the only one that is used in Java is StructureBackend.java. A StructureBackend object is created, and it uses the JNI proxy class to access the C++ code. This is done in a complicated way behind the scenes, and isn't necessary to know in order to use SWIG.

The StructureBackend class encapsulates multiple instances of the StructureImageHandler class inside of it. The StructureImageHandler class is a C++ class that draws structures, and backs not only the structure drawing proxy but the structure drawing text interfaces as well. This ensures that all instances in which a structure is drawn, whether it is in C++ or Java, share the same layout, colorization, and overall look. This is consistent regardless of file format or image type.

The StructureImageHandler instances are built dynamically as needed by feeding a file name string to the proxy class, and then the proxy class creates the image handlers inside itself without exposing those image handlers to SWIG. This allows for a group of many structures to be built inside the proxy, without worrying about memory management issues between the languages. This also stops the Java virtual machine from running out of memory.

The proxy also contains some utility methods to transform structure data into a better format to be accessed by the Java front end, and a method by which the Java interface can pull out all the data for the current structure in string form. The method, called toString, is usually just good for debugging, but it shows a very detailed snapshot of the structure, so it is valuable.

**The Dot Plot Drawing Proxy**

The dot plot drawing proxy consists of the following files. The first four can be found in RNAstructure_java_drawing/SWIG, and the last two can be found in RNAstructure_java_drawing/source/proxy. The first three are written by the developer, and the last three are generated by SWIG.

1. DotPlotBackend.i (SWIG interface file)
2. DotPlotBackend.h (C++ header file for SWIG wrapped class)
3. DotPlotBackend.cpp (C++ implementation file for SWIG wrapped class)
4. DotPlotBackend_wrap.cxx (C++ proxy file generated by SWIG)
5. DotPlotBackend.java (Java class that is used to access wrapped C++)
6. DotPlotBackendProxyJNI.java (Java class that calls native methods from the wrapper class)

Of all these classes, the only one that is used in Java is DotPlotBackend.java. A DotPlotBackend object is created, and it uses the JNI proxy class to access the C++ code. This is done in a complicated way behind the scenes, and isn't necessary to know in order to use SWIG.

The DotPlotBackend class encapsulates a single instance of the DotPlotHandler class inside of it. The DotPlotHandler class is a C++ class that draws dot plots, and backs not only the dot plot drawing proxy but the dot plot drawing text interfaces as well. This ensures that all instances in which a dot plot is drawn, whether it is in C++ or Java, share the same layout, colorization, and overall look. This is consistent regardless of file format or image type.

The DotPlotHandler is built dynamically as needed by feeding a file name string to the proxy class, and then the proxy class creates the handler inside itself without exposing it to SWIG. This allows for a dot plot of any size, with potentially thousands of dots, to be built inside the proxy, without worrying about memory management issues between the languages. This also stops the Java virtual machine from running out of memory.

The proxy also contains some utility methods to transform dot plot data into a better format to be accessed by the Java front end, and a method by which the Java interface can pull out all the data for the current structure in string form. The method, called toString, is usually just good for debugging, but it shows a very detailed snapshot of the dot plot, so it is valuable. The toString method can be called either to describe the plot with its dots or without them. The version that includes the dots, however, can create a very large string that isn't easy to read.

The dot plot proxy doesn't care what type of dot plot file is given to it; it is smart enough to know if a dot plot should be a Dynalign plot, energy plot, or probability plot. Once it knows what kind of plot to display, it figures out the proper dot range and legend type on its own. It can read Dynalign save files, folding save files, partition function save files, or dot plot text files equally well.

**The RNAstructure Java Interface Proxy**

The Java interface proxy consists of the following files. The first four can be found in RNAstructure_java_interface/SWIG, and the last two can be found in RNAstructure_java_interface/source/startup.

1. RNAstructureBackendCalculator.i (SWIG interface file)
2. RNAstructureBackendCalculator.h (C++ header file for SWIG wrapped class)
3. RNAstructureBackendCalculator.cpp (C++ implementation file for SWIG wrapped class)
4. RNAstructureBackendCalculator_wrap.cxx (C++ proxy file generated by SWIG)
5. RNAstructureBackendCalculator.java (Java class that is used to access wrapped C++)
6. RNAstructureBackendCalculatorProxyJNI.java (Java class that calls native methods from the wrapper class)

Of all these classes, the only one that is used in Java is RNAstructureBackendCalculator.java. A RNAstructureBackendCalculator object is created, and it uses the JNI proxy class to access the C++ code. This is done in a complicated way behind the scenes, and isn't necessary to know in order to use SWIG.

The RNAstructureBackendCalculator class contains instances of multiple object-oriented structure prediction classes, including the RNA, HybridRNA, Multilign, and TurboFold objects. It also contains its own TProgressDialog (a class that monitors a calculation's progress) and an associated ProgressMonitor object that allows the Java interface direct access to the percent progress completed.

Some of these objects, particularly the Multilign and TurboFold objects, contain complex data types as parameters or return types for some of their methods. In cases where these methods (for example, the TurboFold constructor) need to be used by the interface proxy, utility methods are created that populate temporary data objects with simple data types, in keeping with the idea of not exposing complex C++ data types to SWIG.

For example, the Multilign constructor takes a two-dimensional vector as a parameter. Clearly, this complex data structure cannot be reliably wrapped by SWIG. So, an additional method was created in the proxy to take in the string vector elements, and populate the vector as a data member inside the proxy. Then, when the run method for Multilign is called in the proxy, the properly populated vector data member can be placed in the Multilign constructor, hidden from both SWIG and Java, with no memory issues. TurboFold follows a similar mechanism when building its constructor data.

Even when there are no complex parameters to make, like vectors or user-defined objects, the complex data objects needed by the proxy (like the RNA object or Dynalign object) are difficult to wrap properly with SWIG as well. So, they follow the same pattern – input the necessary data with a utility method, then use a run method to do the actual calculation with the data structure, completely shielded from SWIG and Java.

The RNAstructure drawing framework is integrated into the Java interface, but the structure and dot plot proxies are not included in the RNAstructureBackendCalculator class. Instead, the independent drawing dialogs are converted into internal windows, and still are allowed to rely on the independent structure and drawing proxies. See the documentation and comments for the DrawingWindow class in RNAstructure_java_interface/source/windows for more on how this is done.

Since the independent structure and dot plot proxies are integrated into the Java interface without being changed, all C++ classes associated with them, either manually written or generated by SWIG, must be linked together into the dynamic library that backs the Java interface.

**Programming Example**

Accessing C++ from Java is very straightforward – for example, in FoldSingleWindow.java (in RNAstructure_java_interface/source/windows):

```
// Create a data structure.

// If an error occurred creating the data structure, show an error

// and return.

String result =

	backend.buildFoldDataStructure( file, isRNA );

	if( !moduleInitialized( result ) ) { return; }
```

The method buildFoldDataStructure takes a single string file name argument and a boolean, which tells if the data structure should be RNA or DNA. When this method is called, the Java JNI class calls the C++ method of the name name. The C++ buildFoldDataStructure method creates an RNA object from the given file name.

C++ methods can be accessed like Java methods, as shown above, once they are wrapped with SWIG.
